# In Vitro Analysis of Tandem Peptides from Human CD5 and CD6 Scavenger Receptors as Potential Anti-Cryptococcal Agents

**DOI:** 10.3390/jof10100667

**Published:** 2024-09-24

**Authors:** Gustavo Mourglia-Ettlin, María Clara González-Porcile, Violeta Planells-Romeo, Antonella Long-Albín, Laura Carrillo-Serradell, Sebastián Miles, Francisco Lozano, María Velasco-de-Andrés

**Affiliations:** 1 Área Inmunología, Departamento de Biociencias (DEPBIO), Facultad de Química, Universidad de la República, Montevideo 11800, Uruguay; gmourglia@higiene.edu.uy (G.M.-E.); claragonzalezporcile@gmail.com (M.C.G.-P.); antonellalong92@gmail.com (A.L.-A.); 2 Unidad Asociada de Inmunología, Instituto de Química Biológica (IQB), Facultad de Ciencias, Universidad de la República, Montevideo 11400, Uruguay; 3 Laboratorio de Inmunología, Instituto de Higiene ‘Prof. Arnoldo Berta’, Universidad de la República, Montevideo 11600, Uruguay; 4 Graduate Program in Biotechnology, Facultad de Ciencias, Universidad de la República, Montevideo 11400, Uruguay; 5 Group of Immunoreceptors of the Innate and Adaptive System, Institut d’Investigacions Biomèdiques August Pi I Sunyer (IDIBAPS), 08036 Barcelona, Spain; planellsi@recerca.clinic.cat (V.P.-R.); lcarrillo@recerca.clinic.cat (L.C.-S.); 6 Graduate Program in Biomedical Research (PROINBIO), Facultad de Medicina, Universidad de la República, Montevideo 11800, Uruguay; 7 Departamento de Desarrollo Biotecnológico, Instituto de Higiene, Facultad de Medicina, Universidad de la República, Montevideo 11800, Uruguay; smiles@fcien.edu.uy; 8 Servei d’Immunologia, Hospital Clínic de Barcelona, 08036 Barcelona, Spain; 9 Departament de Biomedicina, Facultat de Medicina, Universitat de Barcelona, 08036 Barcelona, Spain

**Keywords:** *Cryptococcus*, CD5, CD6, scavenger receptors, peptides, antifungal therapy

## Abstract

*Cryptococcus neoformans* is included in the World Health Organization fungal priority pathogen list, complied to expedite improved research and public-health interventions. The limited number of available antifungal drugs, their associated toxicity, and the emergence of drug-resistant strains make the development of new therapeutic strategies mandatory. Pattern-recognition receptors (PRRs) from the host’s innate immune system constitute a potential source of new antimicrobial agents. CD5 and CD6 are lymphoid members of the ancient scavenger receptor cysteine-rich superfamily (SRCR-SF) which bind pathogen-associated molecular patterns (PAMPs) of fungal and bacterial origin. Evidence supports the concept that such binding maps to 11-mer sequences present in each of their three SRCR extracellular domains. Herein, we have designed synthetic peptides containing tandems of such 11-mer sequences (namely CD5-T and CD6-T) and analyzed their *C. neoformans*-binding properties in vitro. Our results show both inhibitory effects on fungal growth and an ability to impact capsule formation and titanization, two critical virulence factors of *C. neoformans* involved in immune evasion. These effects hold promise for CD5-T and CD6-T peptides as single or adjuvant therapeutic agents against cryptococcosis.

## 1. Introduction

Invasive fungal infections pose a significant threat to global health, affecting over 6.5 million people worldwide with over 50% lethality [1]. The World Health Organization has recently compiled a priority list of fungal pathogens [2], with *Candida albicans*, *Candida auris*, *Aspergillus fumigatus,* and *Cryptococcus neoformans* positioned in the critical group. In particular, *C. neoformans,* along with *C. gattii*, cause around 200,000 cases of meningitis each year, accounting for the most severe form of cryptococcosis [3]. These cryptococcal infections mostly concern immunocompromised individuals, especially those with HIV/AIDS, resulting in high mortality rates, but immunocompetent individuals are also at risk.

*Cryptococcus* spp. exhibit resistance to echinocandins (the latest class of antifungal drugs approved) and can develop resistance to azoles, limiting therapeutic availability to polyenes, particularly amphotericin B in combination with flucytosine and/or fluconazole [2]. Amphotericin B holds significant toxicity and adverse effects, in addition to only being accessible in certain countries. Significant efforts have been made to reduce amphotericin B toxicity through new formulations, with little impact so far [4]. On the other hand, these pathogens produce virulence factors causing host damage and promoting immune response evasion. Indeed, *C. neoformans* accumulates melanin which confers resistance to different stress factors; develops a polysaccharide capsule, avoiding macrophage recognition; secretes degrading proteases and lipases; or undergoes morphological changes such as the formation of large-sized cells named titan cells [5]. These limitations, together with the increasing incidence of resistance, highlight the need for innovative alternative or complementary strategies to available antifungals.

Pathogen sensing and neutralization by receptors from the host’s innate immune system (the so-called pattern-recognition receptors, or PRRs) relies on the recognition of conserved microbial structures not shared by the host but crucial for the pathogen’s viability and virulence (the so-called pathogen-associated molecular patterns, or PAMPs) [6]. PAMP recognition by PRRs thus constitutes an attractive source for developing new antimicrobials. This would be the case for CD5 and CD6, two lymphoid members of the ancient and highly conserved scavenger receptor cysteine-rich superfamily (SRCR-SF) known to act as PRRs for PAMPs of fungal, bacterial, and parasitic origins [7,8,9,10]. CD5 and CD6 are highly related both at the functional and structural levels, pointing to a common gene ancestor [11]. Both receptors are trans-membrane glycoproteins mainly expressed on T cells and a small B cell subset (B1a cells) and characterized by three tandem SRCR extracellular domains and a cytoplasmic tail well adapted for intracellular signal transduction. Studies in CD5 and CD6 knockout mice showed relevant and non-redundant roles in immune defence toward fungal and bacterial infection, respectively [12,13]. These roles depend on the binding of the extracellular SRCR domain to PAMPs from fungi (β-glucans for CD5), bacteria (lipopolysaccharide, lipoteichoic acid, and peptidoglycan for CD6) and parasites (tegumental antigens from *Echinococcus granulosus* for both CD5 and CD6) [7,8,9,10]. Accordingly, the infusion of soluble recombinant human CD5 (rshCD5) and CD6 (rshCD6) ectodomains resulted in prophylactic and therapeutic effects in mouse models of fungal, bacterial, and parasitic infections [9,10,14].

The bacterial PAMP-binding properties of CD6 have been mapped to homologous 11-mer peptide sequences on each of its SRCR domains [15], and similar sequences may account for the fungal PAMP-binding properties of CD5. Here, we have explored whether tandem peptides composed of the three 11-mer sequences found within the SRCR domains of CD5 and CD6 impact fungal pathogenicity, as exemplified by *C. neoformans*’s polysaccharide capsule formation and titanization. Our in vitro results support the value of such tandem peptides as novel potential therapeutic strategies against cryptococcosis. 

## 2. Materials and Methods

### 2.1. Fungal Strain and Growth Conditions

The *C. neoformans* var grubii (serotype A; H99; ATCC 208821; [16]) yeast strain, kindly provided by Dr. Óscar Zaragoza (Mycology Reference Laboratory, Instituto de Salud Carlos III, Majadahonda, Madrid, Spain), was routinely grown overnight at 30 °C with shaking (180 r.p.m) in liquid Sabouraud medium (Condalab, Madrid, Spain).

### 2.2. Peptides Synthesis

**Figure 1:**
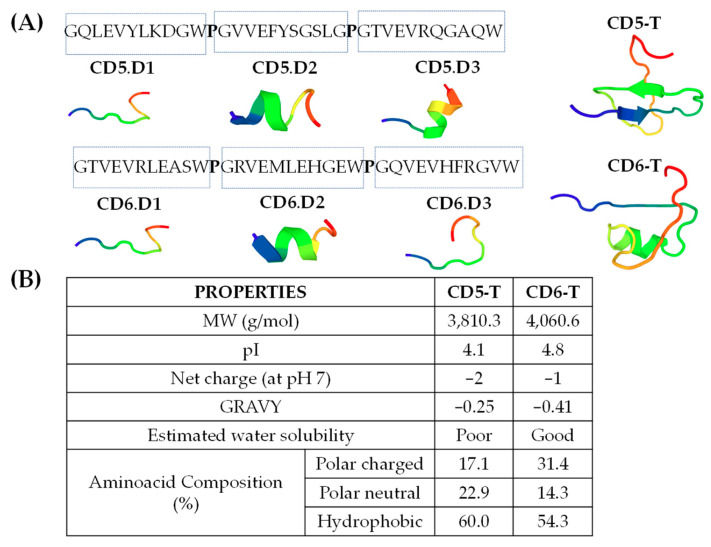
Structural and physicochemical properties of CD5-T and CD6-T peptides. (**A**) Amino acid sequence and schematic 3D representations of CD5-T and CD6-T. (**B**) Physicochemical characteristics and properties of CD5-T and CD6-T peptides. MW (molecular weight), pI (isoelectric point).

CD5 and CD6 tandem peptides (CD5-T and CD6-T; >70% purity) were manufactured by SBS Gentech (Beijing, China) and BCNpeptides (Barcelona, Spain), diluted in deionized water at 2 mg/mL and stored at −80 °C until use. The CD6-T peptide was designed as tandems of microbial binding 11-mer amino acid previously identified in each of the three SRCR extracellular domains of CD6 [15] interspaced by single proline residues (see Figure 1A). The CD5-T peptide was designed based on homologous 11-mer amino acid sequences present in each of the three SCRC extracellular domains of CD5 (see Figure 1A). Three-dimensional models of peptides were obtained using PepFold3 software [17] applying default parameters, while freely available online servers were used for homology studies (LALIGN algorithm) and for the determination of peptides’ physicochemical properties. Aminoacidic compositions were manually calculated.

### 2.3. Assessment of Peptides Biocompatibility

The haemolytic and cytotoxic activity of CD5-T and CD6-T peptides was evaluated as previously reported [18] with slight modifications. Haemolysis was checked on a suspension of erythrocytes (1% *v*/*v*) obtained from female CD1 mice (Instituto de Higiene, Universidad de la República, Montevideo, Uruguay) incubated for 1 h at 37 °C with different peptide concentrations ranging from 0 to 100 μg/mL. Saline solution and sodium dodecyl sulphate (SDS) 10% were used as inducers of 0% (negative control) and 100% (positive control) haemolysis, respectively. Erythrocyte-free supernatants were further measured for absorbance at 560 nm in a microplate reader (Titertek Multiscan Plus; Flow Laboratories, Helsinki, Finland). Results were expressed as the absorbance percentage (% haemolysis) relative to the positive control.

Peptides’ cytotoxicity was evaluated through the MTT (3-(4,5-Dimethyl-2-thiazolyl)-2, 5-diphenyl-2H-tetrazolium bromide) assay applied to mouse macrophage-like RAW264.7 cells following the manufacturer’s instructions (Sigma, St. Louis, MO, USA). To this end, 2 × 10^5^ cells/well were cultured in high-glucose DMEM (Sigma, USA) supplemented with 10% fetal bovine serum (FBS, Capricorn, Düsseldorf, Germany) and antibiotics (100 UI/mL penicillin, Sigma, USA; 100 μg/mL streptomycin, Sigma, USA) for 72 h at 37 °C under a 5% CO_2_ atmosphere in the presence of peptide concentrations ranging from 0 to 100 μg/mL. Afterwards, 50 µL/well of MTT was added and cells were incubated for 4 h at 37 °C to further measure absorbance values at 540 nm in a microplate reader (Titertek Multiscan Plus; Flow Laboratories, Finland). DMEM alone or plus 10% DMSO were used as inducers of 0% (negative control) and 100% (positive control) cytotoxicity. Results were expressed as the absorbance percentage (% viability) relative to the negative control. 

### 2.4. Fungal Agglutination Assays

*C. neoformans* agglutination was performed as in previously reported protocols [15,19]. Briefly, *C. neoformans* (5 × 10^8^ colony-forming units (CFU)/mL) diluted in TTC buffer (50 mM Tris pH 7.5 plus 150 mM NaCl, 0.1% Tween-20, and 1 mM Ca^2+^) were mixed (1:1) with different peptide concentrations (0–200 μg/mL) in U-bottom 96-well microtiter plates. After overnight incubation at 30 °C (w/o shaking), fungal agglutination was examined by light microscopy and scored from absent (−) to maximal (+++) by two independent researchers.

### 2.5. Fungal Viability Assays

To assess the effect of the peptides on fungal growth, *C. neoformans* (1 × 10^3^ CFU/mL) was suspended in liquid Sabouraud medium in the presence of two different concentrations of peptide (10 or 50 µg/mL) in flat-bottom 96-well plates. Sabouraud medium in the absence of peptides was used as the negative control. Fungal growth was determined by measuring the cultures OD at 600 nm during 48 h at 30 °C (with regular shaking) using an Epoch Multiplate Spectrophotometer (BioTek, Winooski, VT, USA).

To assess the effect of the peptides on fungal viability, cultures of *C. neoformans* (0.5 × 10^6^ CFU/mL) were performed in RPMI 1640 medium with L-glutamine plus FBS (10%) for 2 h at 30 °C (w/o shaking) in flat-bottom 96-well plates in the presence of 10 or 50 µg/mL of peptide. For CFU assessment, three serial dilutions (neat, 1:10, and 1:100) in triplicate were then seeded on Sabouraud dextrose agar plates and incubated for 48 h at 30 °C. Similar procedures were performed starting from cultures of *C. neoformans* after the induction of polysaccharide capsule formation or titanization (see below).

### 2.6. Induction of C. neoformans Capsule Formation and Titanization

Polysaccharide capsule formation and fungal cell titanization was induced as previously described [20,21]. Briefly, *C. neoformans* (5 × 10^6^ CFU/mL) was suspended in capsule-inducing medium (10% Sabouraud *v*/*v* in 50 mM MOPS, pH 7.3) and cultured overnight at 37 °C in flat-bottom 96-well plates in the presence of different concentrations of peptides (5–50 µg/mL). Inducing medium and Sabouraud alone were used as positive and negative controls, respectively. For titanization purposes, *C. neoformans* (1 × 10^4^ CFU/mL) was suspended in induction medium (5% Sabouraud *v*/*v*, FBS 5% *v*/*v* and 15 μM NaN_3_ in 50 mM MOPS, pH 7.3) and incubated overnight at 37 °C under a 5% CO_2_ atmosphere in flat-bottom 96-well plates in the presence of different concentrations of peptides (5–50 µg/mL). Inducing medium and Sabouraud alone were used as positive and negative controls, respectively.

Capsule size and titan-like cells’ development was measured after staining with India ink (drawing ink A 523, Pelikan, Hanover, Germany). Samples were observed under an Olympus IX51 microscope using the CellSens Standard 1.12 Imaging and analyzed with ImageJ (v.2.14.0/1.54f). Between 26 and 72 cells were analyzed from different fields which were randomly chosen and photographed. 

### 2.7. Statistics

Continuous variables were analyzed using parametric tests, either Student’s *t*-test or ANOVA, while contingency studies were analyzed by means of Fisher’s exact test. In all cases, differences with *p* < 0.05 were considered statistically significant.

## 3. Results

### 3.1. Design and Characterization of CD5-T and CD6-T Peptides

Homologous 11-mer peptide sequences with putative microbial-binding properties were previously identified within the SRCR ectodomains of CD5 and CD6 [15,22]. Herein, 35-mer peptides (namely CD5-T and CD6-T) were designed by the alignment of such sequences in tandem to explore whether they retained microbial-binding capacity. Each 35-mer peptide was composed of the three 11-mer sequences from each receptor, joined together by a proline (p) residue to minimize structural changes within the 11-mer composing peptides. The results in Figure 1A show the amino acid sequences of CD5-T and CD6-T, as well as their tridimensional (3D)-modelled structures. Three-dimensional models obtained by using the PepFold3 software [17] and applying default parameters of the single 11-mer sequences were used to build up both tandem peptides depicted in Figure 1A.

Three-dimensional models showed that CD6-T displayed high structural conservation around the 11-mer sequences contained in the native protein, with CD5-T suffering more pronounced structural changes. The inner structural order in CD6-T (from its N-terminus in blue to its C-terminus in red) was random coil, followed by an α-helix and a final random coil, in line with the 3D-modelled structures of its individual 11-mer sequences (random coil for CD6.D1, α-helix for CD6.D2, and random coil for CD6.D3). On the contrary, the inner structural order observed in CD5-T was a β-sheet, followed by an antiparallel β-sheet and a final random coil, divergent from its isolated 11-mer sequences (random coil for CD5.D1 and α-helix for both CD5.D2 and CD5.D3).

CD5-T and CD6-T exhibited highly similar physicochemical characteristics (Figure 1B) irrespective of the structural differences observed. CD5-T and CD6-T shared low sequence identity and similarity (42.9 and 62.9%, respectively), but both peptides were acidic according to their pI values and displayed similar negative net charges at pH 7. Additionally, their amino acid composition was quite similar in terms of polar (either charged or neutral) and hydrophobic amino acids content.

**Figure 2:**
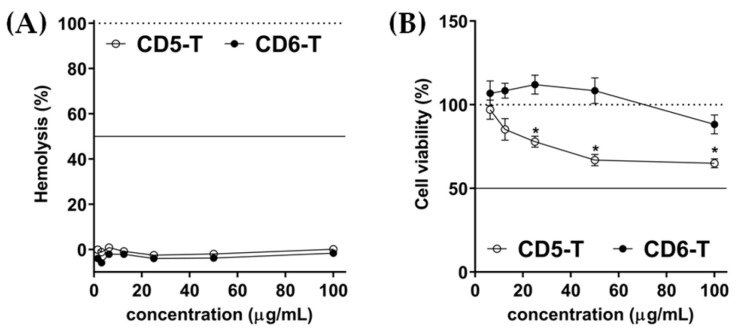
Biocompatibility of CD5-T and CD6-T peptides. (**A**) Haemolytic activity of CD5-T and CD6-T peptides expressed as the percentage referred to saline and 10% SDS-induced haemolysis. The continuous line indicates the HA_50_. (**B**) Cell viability determination by MTT after RAW264.7 cells’ incubation in the presence of CD5-T and CD6-T peptides. DMEM plus 10% (*v*/*v*) DMSO and DMEM alone were used as positive and negative cytotoxic controls, respectively. The continuous line indicates the CA_50_. Results are represented as mean ± SEM of viable cell counts (quadruplicates). Statistical differences were assessed by Student’s *t*-test (*, *p* < 0.05).

CD5-T and CD6-T peptides were synthesized, solubilized in deionized water, and sterile-filtered before storage at −80 °C. The biocompatibility of both peptides was assessed in vitro through haemolytic and cytotoxic activity determinations. Accordingly, biocompatibility assays resulted in HA_50_ and CA_50_ values over 100 µg/mL for both peptides (Figure 2A and Figure 2B, respectively). Nonetheless, significant differences were obtained between peptides regarding cytotoxicity, as lower cell viability values were observed for CD5-T at higher concentrations (Figure 2B). 

### 3.2. In Vitro Effects of CD5-T and CD6-T Peptides on C. neoformans

**Figure 3:**
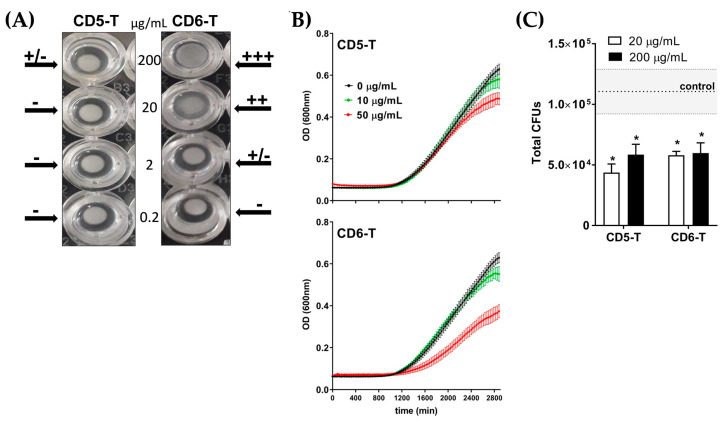
Effect of CD5-T and CD6-T peptides on *C. neoformans* agglutination and viability. (**A**) *C. neoformans* suspensions (5 × 10^8^ CFU/mL) were incubated overnight with increasing concentrations of CD5-T and CD6-T peptides in TTC buffer. Fungal agglutination was arbitrarily scored as −, +/−, +, ++, or +++. Shown is a representative image of two experiments performed. (**B**) Absorbance measurements overtime of *C. neoformans* (1 × 10^3^ CFU/mL) cultured for 48 h in the presence of increasing concentrations of CD5-T or CD6-T peptides. (**C**) Number of viable fungal cells after culturing *C. neoformans* conidia (5 × 10^5^ CFU/mL) for 2 h with vehicle (control) or increasing concentrations of CD5-T and CD6-T peptides. Results are represented as mean ± SEM of CFU (triplicates). The dotted line indicates the control mean values ± SEM marked in grey. Statistical differences were assessed regarding control values by Student’s *t*-test (*, *p* < 0.05).

Agglutination is a simple and efficient mechanism used by some innate immune receptors to avoid pathogen dissemination. At the same time, agglutination provides strong evidence on direct receptor–pathogen interaction. Thus, we first studied the potential of CD5-T and CD6-T peptides to induce fungal agglutination. As illustrated in Figure 3A, the overnight incubation of *C. neoformans* (5 × 10^8^ CFU/mL) with increasing concentrations of both peptides (0–200 µg/mL) showed dose-dependent fungal agglutination. Particularly, CD6-T induced agglutination at almost all tested doses, while CD5-T only exhibited slight agglutination effects (±) at the highest concentration (200 µg/mL).

Once the peptide–microbe interaction was confirmed, the potential effects of CD5-T and CD6-T on fungal viability were further investigated by monitoring *C. neoformans* growth over time in their presence. As illustrated in Figure 3B, growth inhibition was observed for both peptides at the highest tested concentration (50 µg/mL), with CD6-T showing superior effects than CD5-T. Fungal viability was further assessed by monitoring CFU counts in the presence of increasing concentrations of CD5-T and CD6-T peptides. As shown in Figure 3C, the total number of CFUs obtained in the control condition (dotted line) was significantly halved in the presence of CD5-T, irrespective of the concentration assayed. Similar reductions were obtained when *C. neoformans* was cultured in the presence of CD6-T.

### 3.3. In Vitro Interference of CD5-T and CD6-T Peptides on C. neoformans Virulence Factors

**Figure 4:**
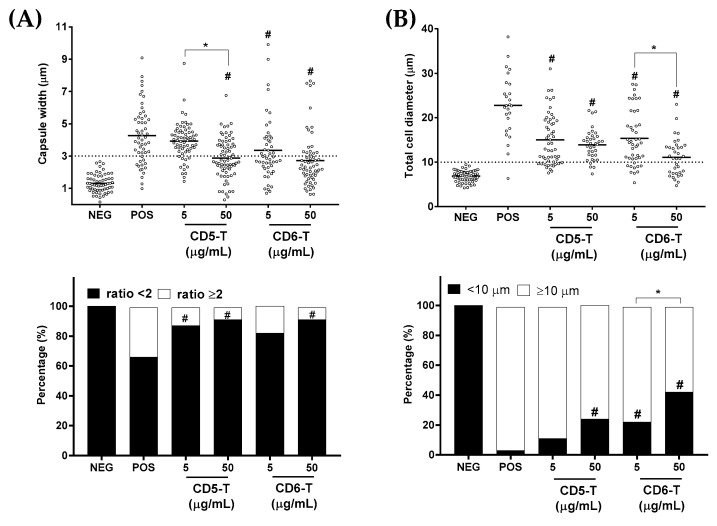
Effect of CD5-T and CD6-T peptides on *C. neoformans* capsule formation and titanization. (**A**) Quantification of fungal capsule width (**top**) and percentages of encapsulated *C. neoformans* cells (**bottom**) after *C. neoformans* were cultured in capsule-inducing conditions in the presence of increasing concentrations of CD5-T or CD6-T peptides. Sabouraud alone was used as negative control. (**B**) Quantification of *C. neoformans* diameter (**top**) and percentages of titan cells (**bottom**) assessed as in (**A**). Continuous variables were analyzed using ANOVA tests, while for contingency analyses, Fisher’s exact test was used. #, significant differences with respect to the positive control. *, significant differences between peptide concentrations. In all cases, differences with *p* < 0.05 were considered statistically significant.

Polysaccharide capsule induction is one of the main virulence factors used by *C. neoformans* for its protection against oxidative stressors and clearance by a host’s phagocytes [20]. By growing *C. neoformans* under capsule-inducing conditions, we observed dose-dependent reductions in both the capsule width (Figure 4A top) and proportion of capsule-positive cells in the presence of CD5-T and CD6-T peptides (Figure 4A bottom). In this regard, the mean positive control values of capsule width (4.27 µm) were significantly reduced in the presence of CD5-T (2.87 µm at 50 µg/mL) and CD6-T (3.35 µm at 5 µg/mL; 2.72 µm at 50 µg/mL) (Figure 4A, top).

Regarding the proportion of capsule-positive cells, similar dose-dependent reductions were observed after comparing the proportion of cells showing a total-diameter-to-body-diameter size ratio ≥2; a characteristic of polysaccharide capsule induction in *C. neoformans* [20]. Thus, while 33.3% of cells showed capsule induction in the positive control condition, this was significantly reduced to 12.1 and 8.3% in the presence of 5 and 50 µg/mL of CD5-T, respectively (Figure 4A, bottom). Similar effects were observed in the presence of CD6-T peptide, 18.0 and 8.3% at the 5 and 50 µg/mL concentrations, respectively, but only the highest concentration reached statistical significance.

Another relevant mechanism of immune evasion by *C. neoformans* is titanization, a process characterized by a huge increase in fungal cell size (normal *C. neoformans* size of 4–6 µm goes to >10 µm values, reaching diameters up to 50–60 µm) [21]. By growing *C. neoformans* under titanization-inducing conditions, dose-dependent reductions in both fungal cell size (Figure 4B top) and the proportion of titan cells were observed in the presence of CD5-T and CD6-T peptides (Figure 4B bottom). An average cell diameter of 22.78 µm versus 6.86 µm was obtained under inducing (positive control) and non-inducing (negative control) conditions, respectively. In the presence of CD5-T, mean cell size values of 15.05 and 13.89 µm were observed for 5 and 50 µg/mL concentrations, respectively. In the case of CD6-T, the mean values were 15.35 and 11.10 µm for 5 and 50 µg/mL concentrations, respectively (Figure 4B top). Additionally, when the proportion of induced titan cells (i.e., cells with a diameter ≥10 µm) was analyzed, significant reductions in comparison with the positive control (96.2%) were observed, either in the presence of 50 µg/mL CD5-T (75.9%) or of 5 and 50 µg/mL CD6-T (77.8 and 57.1%, respectively) (Figure 4B bottom).

**Figure 5:**
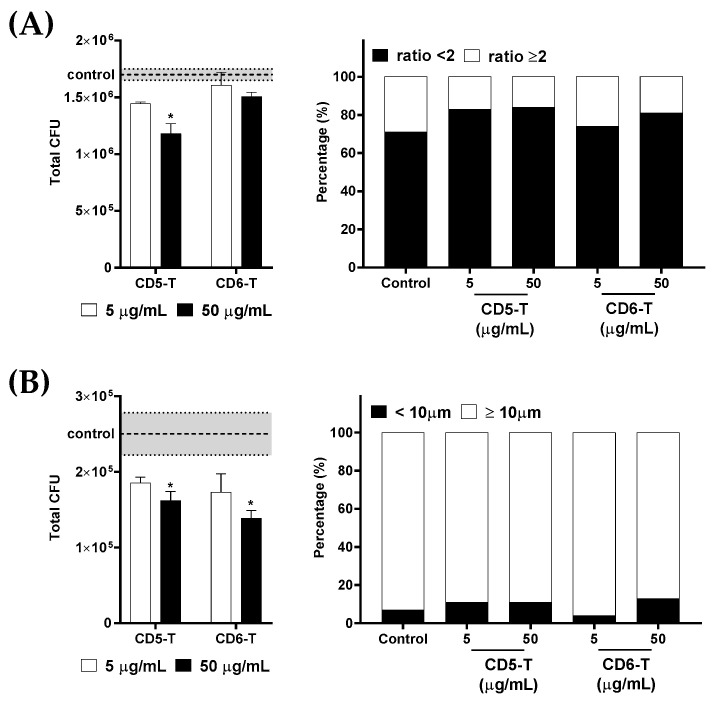
Effect of CD5-T and CD6-T peptides on *C. neoformans* viability after capsule formation and titanization. (**A**) Number of viable *C. neoformans* cells (**left**) after capsule induction and proportion of encapsulated fungal cells after the killing assay (**right**) in the presence of vehicle (control) or increasing concentrations of CD5-T and CD6-T peptides. (**B**) Number of viable *C. neoformans* cells (**left**) after titanization induction and proportion of titan cells after the killing assay (**right**) in the presence of vehicle (control) or increasing concentrations of CD5-T and CD6-T peptides. The dotted line indicates the control mean values ± SEM marked in grey. Continuous variables were analyzed regarding control values using ANOVA tests, while for contingency analyses, Fisher’s exact test was used. *, significant differences with respect to the control condition. In all cases, differences with *p* < 0.05 were considered statistically significant.

Then, the impact of CD5-T and CD6-T on the viability of encapsulated and titanized *C. neoformans* was assessed. CD5-T and CD6-T peptides were added to *C. neoformans* cultures once capsule formation or titanization was induced. After overnight incubation, fungal viability was assessed by CFU counts. As illustrated in Figure 5A,B, dose-dependent deleterious effects of CD5-T and/or CD6-T peptides were observed on the viability of both encapsulated and titanized *C. neoformans*. CD5-T was able to reduce the viability of *C. neoformans* after capsule induction in a concentration-dependent manner (Figure 5A, left), while CD6-T’s inhibitory effects did not reach statistical significance at the tested concentrations. Similarly, CD5-T and CD6-T peptides showed dose-dependent deleterious effects on the viability of titanized cells (Figure 5B, left).

Finally, the viable fungal cells from overnight cultures were morphologically characterized. No significant preference of CD5-T and CD6-T peptides for killing encapsulated versus non-encapsulated (Figure 5A, right) or titanized versus non-titanized *C. neoformans* cells was observed (Figure 5B, right). Thus, CD5-T and CD6-T peptides showed fungal killing activity regardless of the morphological state of *C. neoformans*. 

## 4. Discussion

Invasive fungal infections constitute an important challenge to public health due to their growing prevalence, high morbi/mortality, and escalating antimicrobial resistance [1]. However, the need for new therapeutic alternatives is hindered by the similarities shared between fungi and mammalian cells, including metabolic and cell membrane components. This challenge can be overcome through strategies based on PRRs from the innate immune system, since they bind to and neutralize conserved pathogenic microbial structures (PAMPs) not shared by the host. 

The therapeutic potential of immune-related compounds has long been explored [23]. The high economic and time costs associated with mammalian protein production align with the interest in the development of synthetic peptides. Antimicrobial peptides provide important advantages such as safety, high specificity, and a low probability of generating microbial resistance [24]. Thus, during the past decade, multiple peptide agents with different characteristics have proved efficient against a large variety of fungal pathogens [25]. *C. neoformans* has not been left out of this race, with effective candidates exhibiting a broad spectrum of mechanisms of action [26,27,28]. 

In the present report, we have leveraged the PAMP-binding properties of lymphoid-specific scavenger receptors, namely CD5 and CD6, to explore a cost-effective antifungal alternative based on synthetic tandem peptides that retain the properties of native proteins. By means of CD5- and CD6-deficient mice, it has been demonstrated that both lymphocyte receptors are integral and non-redundant components of the host’s immune defence against fungal and bacterial infections [12,13]. Accordingly, the infusion of soluble recombinant forms of CD5 (rshCD5) and CD6 (rshCD6) showed antifungal and antibacterial, but also antiparasitic, properties in in vivo models [10,14,29]. Exploration of the molecular basis of PAMP recognition by CD6 allowed for the identification of homologous 11-mer peptide sequences located in each of its three SCRC ectodomains [15]. The overall structural and functional homology between CD5 and CD6 served as the starting point for the design of 35-mer peptides (CD5-T and CD6-T) encompassing all three conserved 11-mer PAMP-binding motifs present in both receptors in tandem. As expected, CD5-T retained the fungal-binding properties previously reported for rshCD5 [29] as shown by its killing activity on *C. neoformans*. Unexpectedly, similar fungal-binding properties were shared by CD6-T, since rshCD6 interaction with saprophytic (*Sacharomyces pombe*) but not pathogenic (*C. albicans* and *C. neoformans*) fungal species was the sole antifungal ability reported [7]. This unexpected finding should still not contradictory with the notion that PRR in general and SRCR-SF receptors in particular are multi-ligand receptors recognizing a diverse range of apparently different PAMP structures [11]. 

The tandem peptides designed exhibit similarities from a physicochemical standpoint (e.g., net charge, acidity, and hydrophobicity), but also significant differences from a structural perspective. These may lie behind the functional differences observed against *C. neoformans*. Thus, while both peptides showed similar in vitro antifungal activity regardless of the morphological state of *C. neoformans*, CD6-T was more efficient in interfering with fungal growth, capsule formation, and titanization. Such a difference may probably derive from the greater ability of CD6-T to interact with this fungal pathogen, as we have observed higher aggregation power compared to CD5-T.

A remaining question is whether the antifungal activity observed for CD5-T and CD6-T against *C. neoformans* can be extended to other fungal species. The only data available are our own preliminary results (Velasco-de Andrés M et al., unpublished ) in this regard showing the significant inhibition of *C. albicans* and *C. aureus* growth by both CD5-T and CD6-T peptides at the highest concentration tested (50 µg/mL). Another important question relates to the fungal structure (s) recognized by CD5-T and CD6-T peptides. Again, the only available information comes from previous studies with rshCD5 and rshCD6 proteins which demonstrated significant binding to β-glucans, a broadly distributed structural component of fungal cell walls, only for rshCD5 [7]. So, a detailed exploration of the binding properties of CD5-T and CD6-T peptides to a broad panel of fungal PAMP structures is mandatory. Such studies must include glucuronoxylomannan (GXM) and glucuronoxylomannogalactan (GXMGal) polysaccharides, which are the main components of *C. neoformans* capsules [5].

## 5. Conclusions

In conclusion, our results constitute the first proof of concept for the study of CD5-T and CD6-T as alternative or adjunctive agents to the currently available antifungal drugs against cryptococcosis. The main antifungal effects induced by these tandem peptides were reductions in fungal viability and virulence factors (i.e., capsule development and titanization). In fact, our own preliminary in vivo studies support the potential efficacy of CD6-T infusion in a mouse model of nasal *C. neoformans* infection (Velasco-de Andrés M. et al., unpublished). The use of CD5-T and CD6-T as therapeutic agents is supported by the preliminary safety studies presented here, where no haemolytic or cytotoxic activity against mouse erythrocytes and macrophage-like RAW264.7 cells was observed. 

## Data Availability

The original contributions presented in the study are included in the article; further inquiries can be directed to the corresponding author.
